# Risk factors for bradypnea in a historical cohort of surgical patients receiving fentanyl-based intravenous analgesia

**DOI:** 10.1186/s40981-018-0186-x

**Published:** 2018-06-13

**Authors:** Takashi Shiota, Hideaki Kawanishi, Satoki Inoue, Junji Egawa, Masahiko Kawaguchi

**Affiliations:** Department of Anesthesiology and Division of Intensive Care, Nara Medical University, 840 Shijo-cho, Kashihara, Nara 634-8522 Japan

**Keywords:** Bradypnea, Respiratory rate monitoring, Acoustic monitoring, Postoperative respiratory deterioration, Postoperative opioid

## Abstract

**Introduction:**

The use of both pulse oximetry (SpO_2_) and respiration rate (RR) monitoring is recommended to prevent the development of respiratory deterioration, particularly after extubation and narcotic analgesic use for pain management. In this study, we investigated the factors contributing to the development of bradypnea in surgical patients receiving fentanyl-based intravenous analgesia after general anesthesia.

**Methods:**

This study involved a historical chart review of postoperative patients outside an intensive care unit setting. We divided the patients according to the data collected during the first hour postoperatively, into those developing bradypnea (RR < 8 breaths per min for > 2 min) and those with normal RR under oxygen administration. We defined oxygen desaturation as SpO_2_ < 90% for > 10 s. We calculated the effect-site concentrations for fentanyl at the end of surgery and 1 h postoperatively using custom-made software based on chart records. A multivariable analysis was used to determine bradypnea-associated explanatory factors.

**Results:**

For the final analysis, we included 258 patients. We detected bradypnea in 125 patients (48%) and oxygen desaturation in 46 patients (18%). We found no difference in the effect-site fentanyl concentrations between patients with and without bradypnea. The logistic regression model revealed that liver dysfunction [odds ratio (OR), 2.918; 95% confidence interval (CI), 1.329–6.405], renal dysfunction (OR, 0.349; 95% CI, 0.128–0.955), and smoking history (OR, 0.236; 95% CI, 0.075–0.735) were independently associated with bradypnea. We found similar incidences of oxygen desaturation between the groups.

**Conclusions:**

Bradypnea was observed in 48% of postoperative patients receiving fentanyl-based intravenous analgesia under oxygen therapy. According to our results, impaired liver function associated positively, whereas smoking history associated negatively with its development. Renal dysfunction was paradoxically associated with less incidence of bradypnea.

## Background

Patients in the immediate postoperative period may develop respiratory deterioration because of residual muscular relaxants and anesthetics or because of postoperative analgesics, which may induce hypoxemia and hypercapnia. Under such settings, pulse oximetry (SpO_2_) is widely used as a continuous, noninvasive oxygenation monitoring method. However, even with hypoventilation, oxygen saturation is usually maintained in postoperative patients receiving supplemental oxygen, which is frequently used in the early postoperative period. Therefore, postoperative oxygen therapy may mask the diagnosis of hypoventilation caused by bradypnea or hypopnea [1, 2]. Thus, the SpO_2_ monitor alone may not be enough to detect hypoventilation in patients receiving postoperative oxygen therapy [1, 2]. However, lethal hypercapnia because of the delayed detection of bradypnea is possible despite the presence of normal oxygen saturation [2]. In addition, the importance of continuous respiration rate (RR) monitoring under various clinical conditions is being increasingly recognized [2]. Thus, the Anesthesia Patient Safety Foundation (APSF) recommends both SpO_2_ and RR monitoring, particularly after extubation and narcotic analgesic use for pain management [2, 3].

Continuous RR monitoring is necessary for spontaneously breathing patients at risk for ventilatory depression; however, easy and reliable methods for continuous RR monitoring have not yet been established. Numerous factors influence the accuracy of thoracic impedance measurements. For example, this technology may rely on chest movements such as those during obstructive apneic episodes in which the chest wall continues to move as the patient attempts to breathe [4, 5]. Capnometry requires a nasal cannula or a face mask that may be unpleasant or can be removed by patients. Occasionally, hypoventilation may paradoxically cause the end-tidal CO_2_ (EtCO_2_) to decrease because of the inadequate mixing of alveolar gas [6, 7]. Therefore, a new more reliable continuous RR monitoring device is needed. The application of a noninvasive respiratory monitoring device using adhesive sensors with an integrated acoustic transducer positioned on the patient’s throat (Rainbow Acoustic Monitoring™, Masimo, Irvine, CA, USA) is a relatively new method of ventilation monitoring, and it provides an accurate estimation of RR in the post-anesthesia and intensive care units and in the emergency room [8–11]. Because of its simple and convenient nature, we have implemented a pulse oximeter with an acoustic RR (RRa) monitoring device (Rad-87™, version 7805, Masimo) in postoperative settings outside of the intensive care unit for the early detection of respiratory deterioration.

In our institute, we use fentanyl-based intravenous analgesia (IV-fentanyl) as a postoperative analgesic option. When IV-fentanyl is used, we conduct both the SpO_2_ and the RR monitoring with Rad-87 according to APSF recommendations. In this study, we retrospectively investigated the incidence of bradypnea in the immediate postoperative period with IV-fentanyl and identified factors related to its development. In addition, we retrospectively calculated effect-site concentrations of fentanyl using simulation computer software based on fentanyl administration profiles from anesthesia charts, and we investigated whether effect-site concentrations of fentanyl affected the incidence of postoperative bradypnea.

## Methods

The Nara Medical University Institutional Review Board approved access to the data stored on the RRa device pulse oximeter and the review of clinical charts of postoperative patients with continuously monitored RRa (Chairperson Prof. N Kurumadani, Nara Medical University, Kashihara, Nara, Japan, 634–8522, Japan), approval No. 1057 on 08–21-2015. The requirement for written informed consents for this historical study was waived.

### Standard perioperative patient treatment

We excluded cases requiring intensive care or similar treatment from RRa monitoring because mechanical ventilation or manual RR monitoring, such as intermittent auscultation every 1–2 h, was required in these cases. We applied RRa monitoring to extubated surgical patients outside of intensive care units. The application was limited to elective cases, partially due to a shortage of RRa devices. We considered patients who underwent craniotomy, thyroidectomy, spinal surgery, laparotomy, laparoscopic surgery, or major orthopedic surgery as candidates for RRa monitoring.

The methods of anesthetic induction and maintenance, as well as those for tracheal intubation, were not standardized for each patient. We used sevoflurane or propofol to maintain anesthesia and fentanyl or remifentanil for analgesia. Rocuronium was used for neuromuscular blockade and sugammadex for the reversal of the neuromuscular blockade, after evaluation of the status of the neuromuscular blockade using a nerve stimulator. The attending anesthetist managed the fluids at their discretion and performed transfusions if necessary. As mentioned, we provided postoperative analgesia with IV-fentanyl (0.2–0.8 μg/kg/h) using a commercially available patient-controlled analgesia (PCA) device (Coopdech Syrinjector PCA Device™, Daiken Medical, Osaka City, Japan). The PCA bolus sizes and lockout timings were set at 0.2–0.8 μg/kg and 10 min, respectively. Low-dose droperidol (1.25–2.5 mg/day) was combined with PCA administration. In the immediate postoperative period (1–2 h postoperatively), caregivers administered each PCA bolus, depending on the individual patient situation. Each time a PCA bolus was given, it was recorded in the medical chart. Upon discharge from the operating room, an adhesive acoustic respiration sensor (RAS-125™ or RAS-125™ rev C, Masimo) and an oximetry sensor (LNCS Adtx, Masimo) were placed on the patient’s neck and finger, respectively, and were connected to an RRa device pulse oximeter to monitor and regularly record the RR, SpO_2_, and pulse rate (PR).

The acoustic sensor was placed on either side of the larynx, above the thyroid cartilage, and below the jaw line according to the manufacturer’s instructions. Oxygen (3–5 l/min) was administered through an oxygen mask, according to our institutional standard practice. Patients were then directly transferred from the operating room to a general surgical ward. The general hospital setting had a nurse-to-patient ratio of 1:7 and comprised a surgical population undergoing miscellaneous surgeries with opioid-based PCA. The nurses at our institute were trained to deliver standard care and to pat a patient on the shoulder or chest while calling or orally encouraging them to breathe deeply in the case of an RRa or SpO_2_ alarm. However, the nurses seldom adjusted the oxygen delivery rate. Patients were continuously monitored with an RRa device containing a pulse oximeter as per the standard of care, and the monitored data were stored in the internal memory. We set RR, SpO_2_, and PR alarms at RR < 8 or > 30 breaths per min, < 90% SpO_2_ with supplemental oxygen, or PR > 130 or < 50 beats per min, respectively, according to the institutional protocol. The RRs and SpO_2_ in the Rad-87 were calculated by each 10-s interval moving average every 2 s and by each 8-s interval moving average every 1 s, respectively. The RRa device pulse oximetry data were temporarily and automatically stored in an internal memory for up to 72 h with a 2-s resolution. Every time that Rad-87 monitoring was terminated, clinical engineers transferred the data to a storage device using downloaded software (TrendCom ver. 3460, Masimo) and saved the file at our institute.

### Data handling and statistical analysis

**Fig. 1:**
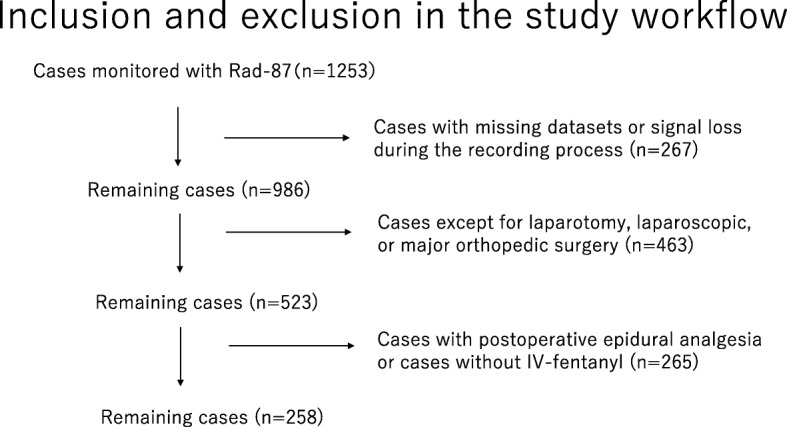
Study workflow. IV-fentanyl, fentanyl-based intravenous analgesia

We collected data from May 1, 2012, to October 31, 2013. During this period, 1253 adult surgical patients were monitored with an RRa device pulse oximeter. We extracted the cumulatively stored data and used the data collected during the first postoperative hour for analyses. We used sophisticated artifact detection algorithms to identify invalid artifact periods in the stored data. That way we confirmed data reliability after prescreening the raw data. We excluded (1) cases with missing datasets or signal loss during the recording process (*n* = 267); (2) cases undergoing any procedures other than laparotomy, laparoscopic, or major orthopedic surgeries (*n* = 463) (because generally IV-fentanyl was not provided to these cases according to the institutional protocol); and (3) cases with postoperative epidural analgesia or without IV-fentanyl use (*n* = 265). In the end, we used the data from 258 patients for this study (Fig. 1).

In addition, we extracted perioperative data from the patients’ clinical charts. On the basis of the analyzed data, we defined an oxygen desaturation event as that with an SpO_2_ < 90% for > 10 s and bradypnea as an RR < 8 breaths per min for > 2 min, based on arbitrary local rules [12]. Next, we calculated the fentanyl effect-site concentrations at the end of surgery and 1 h postoperatively using BeConSim (owned and produced by Kenichi Masui, Anesthesiology, National Defense Medical College, http://www.masuinet.com/), based on the intraoperative administration profiles of fentanyl and usages of IV-fentanyl including PCA counts.

Subsequently, we divided the patients into the following two groups: (1) the bradypnea group and (2) the normal RR group. Initially, we used univariate logistic regression analyses to identify candidate factors associated with bradypnea. Variables such as age, gender, height, body weight, body mass index (BMI), % vital capacity (%VC), forced expiratory volume % in 1 s, American Society of Anesthesiologists’ physical status classification, sleep apnea syndrome history, renal dysfunction, liver dysfunction, hypertension, asthma, chronic obstructive pulmonary disease, hyperlipidemia, diabetes mellitus, ischemic heart disease, chronic heart failure, smoking, hemodialysis, anesthesia method, surgical site, surgery and anesthesia duration, fluid balance, transfusion balance, total fluid balance (fluid balance + transfusion balance), IV-fentanyl infusion rate, PCA usage count, and effect-site concentrations of fentanyl at the end of surgery and 1 h postoperatively are presented as adjusted odds ratios (ORs) for the development of bradypnea with 95% confidence intervals (CIs). Regarding each medical history, only the presence (or absence) of illness was considered but not the disease severity. In short, the presence or absence of disease was based upon the results of preoperative abnormal tests, or history-based documents. We used explanatory factors exhibiting a significant univariate association (*p* < 0.20) with bradypnea to construct a forced-entry multivariate logistic regression model and presented them as adjusted ORs with 95% CIs. Interactions between the variables were systematically searched, and collinearity was considered when *r* or rho was > 0.8, according to the Pearson or Spearman’s coefficient matrix correlation. However, we forced the calculated fentanyl effect-site concentrations into the final model regardless of their statistical significance in the univariate analysis based on our hypothesis. We assessed the discrimination of the final model for bradypnea based on the likelihood ratio test. We used the Hosmer–Lemeshow statistical method to calibrate our model. The area under the receiver operating characteristic (ROC) curve was computed as a descriptive tool for measuring model biases. In addition, we compared the incidence of oxygen desaturation and delivered oxygen flow between the groups. Data are expressed as mean (standard deviation) for normally distributed variables and as median (interquartile range) for non-Gaussian distributed ones. The comparison of two means was performed using Student’s *t* test and that of two medians or two proportions using the Mann–Whitney *U* test and the *χ*^2^ test or Fisher’s exact method, respectively. We used the SPSS statistical package (SPSS for Windows, Version 24.0. Chicago, SPSS) to perform all the analyses.

## Results

No patients died or required a code-blue call or intensive care during the study period. Moreover, there were no reports of critical complications. To investigate the overall incidence of bradypnea, we used data from 258 patients with complete datasets and without signal loss during the recordings. The defined bradypnea (RR < 8 breaths per min for > 2 min) was observed in 125 (48%) patients. The median number of times the alarm rang for patients with bradypnea as per the preset alarm range was three (IQR, 1–12). Incidentally, no patients showed abnormally increased RRs (RRs > 30 beats per min for > 2 min). We observed oxygen desaturation in 46 (18%) patients. Patient data and perioperative characteristics comparisons between the bradypnea and normal RR groups can be found in Table 1. Univariate logistic regression analyses detected only two significant factors, namely, histories of liver dysfunction (*p* = 0.023) and smoking (*p* = 0.022). We extracted data on BMI; %VC; liver dysfunction, renal dysfunction, hypertension, and smoking histories; total fluid balance; and duration of anesthesia as candidates associated with bradypnea development based on the results of the univariate logistic regression analyses. Our logistic regression model showed that liver dysfunction (OR, 2.918; 95% CI, 1.329–6.405), renal dysfunction (OR, 0.349; 95% CI, 0.128–0.955), and smoking history (OR, 0.236; 95% CI, 0.075–0.735) were independently associated with the development of bradypnea (Table 2). However, simulated fentanyl effect-site concentrations did not show any association with the model. The discrimination of the final model assessed by the likelihood ratio test was significant (*p* = 0.003). Moreover, the Hosmer–Lemeshow statistic did not reject a logistic regression model fit (*p* = 0.258). The explanatory model based on these variables had an area under the ROC curve of 0.681 (95% CI, 0.620–0.737). We performed post hoc power calculations for these multivariable logistic regression models using 10 variables. We followed standard methods to estimate the sample size for multivariate logistic regression, with at least 10 outcomes needed for each included independent variable [13]. With a 48% (125/258) incidence of bradypnea in the overall study population, we required the data from 208 patients monitored using the RRa device pulse oximeter to appropriately perform a multivariate logistic regression with 10 variables. Thus, our sample size was sufficient to build the model.

**Table 1:** Demographic and clinical characteristics and results of univariable logistic regression analyses for development of abnormal RRs

	Bradypnea (*n* = 125)	Normal RR (*n* = 133)	Odds	95% CI	*P* value
Age (year)	63.1 (13.4)	63.6 (13.3)	0.973(/10 years)	0.809–1.169	0.771
Height (cm)	157.9 (8.3)	157.9 (10.2)	1.130(/10 cm)	0.753–1.537	0.976
Weight (kg)	57.2 (11.9)	59.1 (12.9)	0.841 (/10 kg)	0.666–1.061	0.214
Sex (F/M)^a^	85/40	78/55	1.499	0.900–2.496	0.124
BMI	22. 9 (4.1)	23.7 (4.5)	1.004	0.992–1.017	0.149
%VC	107.2 (19.0)	104.6 (17.6)	1.082 (/10%)	0.946–1.239	0.249
FEV1.0%	77.9 (10.0)	79.5 (11.1)	0.869 (/10%)	0.686–1.102	0.245
ASA status (I/II/III)	30/87/8	23/98/12	0.703	0.435–1.135	0.344
Surgical site^b^					
Upper abdominal laparotomy	13	10	0.831	0.309–2.231	0.731
Lower abdominal laparotomy	44	60	1.473	0.755–2.875	0.257
Laparoscopic surgery	41	38	1.001	0.497–2.017	0.998
Orthopedic surgery	27	25	1(reference)	1	1
Anesthesia (propofol/volatile)^c^	36/89	43/90	0.847	0.498–1.440	0.590
IV-fentanyl basal dose (μg/h)	22.0 (7.3)	22.6 (6.7)	0.989	0.955–1.025	0.554
PCA usage count	1 [1–1]	1 [1–1]	1.142	0.742–1.759	0.546
Fluid balance (ml)	1850 [210–6142]	1895 [− 450–7245]	0.988(/100 ml)	0.969–1.008	0.238
Transfusion balance (ml)	−55 [− 1514–635]	−75 [− 2165–762]	0.977(/100 ml)	0.918–1.040	0.467
Total fluid balance (ml)	1740 [410–4702]	1832 [− 500–6525]	0.980(/100 ml)	0.957–1.003	0.091
Liver dysfunction (Y/N)	25/100	13/120	2.308	1.122–4.745	0.023
Renal dysfunction (Y/N)	7/118	15/118	0.467	0.184–1.186	0.121
Hypertension (Y/N)	84/41	77/56	0.671	0.404–1.115	0.157
Asthma (Y/N)	2/123	2/131	1.065	0.148–7.678	1.000
COPD (Y/N)	9/116	5/128	1.986	0.647–6.098	0.277
Hyperlipidemia (Y/N)	8/117	14/119	0.581	0.235–1.437	0.270
Diabetes mellitus (Y/N)	15/110	18/115	0.871	0.418–1.814	0.852
Ischemic heart disease (Y/N)	5/120	6/127	0.882	0.262–2.966	1.000
Heart failure (Y/N)	1/124	1/132	1.065	0.066–17.204	1.000
Smoking (Y/N)	5/120	16/117	0.305	0.108–0.859	0.022
Hemodialysis (Y/N)	1/124	4/129	0.260	0.029–2.359	0.371
Fentanyl 0 h (ng/ml)	1.1 (0.3)	1.1 (0.3)	0.884	0.452–1.726	0.719
Fentanyl 1 h (ng/ml)	1.0 (0.2)	1.0 (0.3)	0.707	0.284–1.759	0.457
Duration of surgery (min)	204 [32–1202]	241 [51–594]	0.995(/10 min)	0.978–1.012	0.336
Duration of anesthesia (min)	268 [81–1004]	315 [89–712]	0.986(/10 min)	0.968–1.004	0.104

Bradypnea change was defined as an RR < 8 breaths per min in 2 min or longer

*RR* respiratory rate, *BIM* body mass index, *%VC* % vital capacity, *FEV1.0%* forced expiratory volume % in 1 s, *Propofol* propofol-maintained anesthesia, *Volatile* volatile anesthetics-maintained anesthesia, *IV-fentanyl* fentanyl-based intravenous analgesia, *PCA* patient-controlled analgesia, *Total fluid balance* fluid balance + transfusion balance, *COPD* chronic obstructive pulmonary disease, *Fentanyl 0 h* effect site concentrations of fentanyl at the end of surgery, *Fentanyl 1 h* effect site concentrations of fentanyl 1 h postoperatively

^a^Male sex is defined as the reference level

^b^Orthopedic surgery is defined as the reference level

^c^Volatile anesthetics is defined as the reference level

**Table 2:** Results of multivariable logistic regression analyses for development of bradypnea

	Odds	95% CI	*P* value
Sex^a^	1.401	0.796–2.464	0.242
BMI	0.949	0.889–1.013	0.114
Total fluid balance	0.990 (/100 ml)	0.955–1.025	0.560
Liver dysfunction	2.918	1.329–6.405	0.008
Renal dysfunction	0.349	0.128–0.955	0.040
Hypertension	0.669	0.389–1.151	0.146
Smoking	0.236	0.075–0.735	0.013
Duration of anesthesia	0.995 (/10 min)	0.967–1.024	0.739
Fentanyl 0 h (ng/ml)	1.467	0.337–6.379	0.610
Fentanyl 1 h (ng/ml)	0.442	0.052–3.780	0.456

*BMI* body mass index, *Fentanyl 0 h* effect site concentrations of fentanyl at the end of surgery, *Fentanyl 1 h* effect site concentrations of fentanyl 1 h postoperatively

^a^Male sex is defined as the reference level

We found no significant differences in the incidence of oxygen desaturation [21/125 (16.8%) vs. 25/133 (18.8%), *p* = 0.746] and delivered oxygen flow [3 (3–3) l/min vs. 3 (3–3) l/min, *p* = 0.588] between the bradypnea and normal RR groups.

## Discussion

Bradypnea and oxygen desaturation under IV-fentanyl management were respectively observed in 48 and 18% of patients, whose RR was monitored using RRa devices. In terms of the causative factors for postoperative bradypnea, our study results showed that a history of liver dysfunction may increase the possibility of developing bradypnea; contrarily, histories of renal dysfunction and smoking may decrease the likelihood of the event, although simulated effect-site concentrations of fentanyl did not show any associations.

Overdyk et al. reported that although the incidence of oxygen desaturation was 12%, bradypnea was 41–58% in patients receiving PCA therapy with continuous monitoring using both pulse oximetry and noninvasive capnography [14]. Those results are consistent with ours, which suggest that considerable respiratory depression events can happen even without oxygen desaturation when parenteral opioids are used in the postoperative period. In the current study, the incidences of oxygen desaturation were similar between the bradypnea and normal RR groups. This may be due to the effectiveness of the caregiver treatment under RRa monitoring, in which the nurses at our institute delivered standard care and encouraged patients to breathe deeply and faster when alarms rang. Generally, bradypnea occurs prior to the development of oxygen desaturation in patients receiving supplemental oxygen [1, 2]. Therefore, it is likely that development of desaturation was prevented in many cases because the caregivers stimulated respiration with supplemental oxygen as soon as a bradypnea event became manifest. However, approximately 20% of the patients showed oxygen desaturation in the group without bradypnea, although the absence of bradypnea generally suggests a stable respiratory status. This may imply that the absence of bradypnea does not guarantee that such patients will not develop oxygen desaturation under our monitoring setting. These patients may have temporarily developed abnormal conditions, such as impaired pulmonary function or morbid pulmonary conditions.

Our study indicates that a history of liver dysfunction can be associated with the development of bradypnea. However, it is unlikely that liver dysfunction per se directly acted as a depressant of respiration because our population did not include patients with severe liver failure. In addition, the pharmacokinetics of phenylpiperidine opioids such as fentanyl, sufentanil, and remifentanil appear to be rarely affected by hepatic disease [15]. Moreover, the opioids fentanyl, sufentanil, and remifentanil may be the best choices in liver insufficiency cases [16]. However, because fentanyl is indeed metabolized in the liver, its pharmacokinetics may vary depending on the different stages of liver disease. Although the fentanyl effect-site concentrations calculated by BeConSim were similar between the groups, it is possible that the actual concentrations in the bradypnea group were significantly higher. This possibility cannot be completely ruled out, but this remains a matter of speculation. Therefore, the administration profiles of IV-fentanyl may have to be reconsidered in cases of liver dysfunction. Meanwhile, renal dysfunction decreased the development of bradypnea. The liver metabolizes fentanyl with a high hepatic clearance. However, in general, renal impairment is one of the factors contributing to opioid-induced respiratory depression in pain management studies [17]. It is possible that this fact affects the anesthesiologists’ judgment, leading them to consider reducing the dose of fentanyl administered to patients with renal dysfunction to avoid a relative overdose. Therefore, it is possible that the under-administration of fentanyl resulting from the attending physician’s judgment resulted in lower bradypnea events in renal dysfunction cases. In fact, our post hoc analysis revealed that patients with renal dysfunction were given a similar dose of fentanyl intraoperatively [1.66 μg/kg/h (0.74) vs. 1.80 μg/kg/h (0.57), *p* = 0.386] but a significantly lower basal dose of postoperative IV-fentanyl [0.34 (0.08) μg/kg/h vs. 0.39 (0.10) μg/kg/h, *p* = 0.024] than those in patients without renal dysfunction. However, the number of patients with renal dysfunction in the whole population was low. Therefore, the means of simulated fentanyl concentrations may not have been affected significantly.

A history of smoking significantly suppressed the development of bradypnea. It has been consistently shown that by altering the metabolism of opioids, smoking increases the requirements for opioids [18]. Smokers undergoing coronary artery bypass grafting require increased fentanyl concentration [19]. Thus, this result may reflect the tolerance to opioids by smokers. However, the association between the analgesic effects of fentanyl and the absence of bradypnea in smokers remains unclear because records of pain assessment during the study period were unavailable.

We are aware of the limitations in our study. First, because of the retrospective study design, we may have overlooked important confounding factors related to the development of bradypnea. Another potential limitation is that bradypnea or oxygen desaturation may have occurred during the artifact periods detected by the Masimo algorithms. However, a system status alert would have made the nurses aware of the problem, and this probably did not alter the results of our study. Also, when simulated concentrations of fentanyl were calculated, we took into consideration the PCA usage count by caregivers. However, we may have missed counting records, although we believe there were few missed records if any regarding the PCA counts, because the immediate postoperative patients generally receive close attention. On the other hand, the software used for the simulation of IV-fentanyl concentrations in this study, BeConSim, was based on Shafer’s pharmacokinetic model. But the software did not have any option to adjust the simulation based on liver or renal functions. In addition, BeConSim was developed by a private investigator, and its simulation may be less reliable than those by company-based software programs. Finally, postoperative bradypnea and desaturation are serious postoperative adverse events during the initial 24 h. However, for our study, we focused on just the first hour after surgery, and the limited follow-up period is one of the study limitations.

## Conclusions

Bradypnea developed in 48% of the postoperative patients receiving IV-fentanyl under oxygen therapy. Impaired liver function associates positively, whereas smoking history associates negatively with its development. In addition, renal dysfunction was paradoxically associated with less incidence of bradypnea. A randomized controlled trial for a more controlled population is warranted.

## Abbreviations

%VC: % Vital capacity
APSF: Anesthesia Patient Safety Foundation
BMI: Body mass index
CI: Confidence interval
EtCO_2_: End-tidal CO_2_
FEV1.0%: Forced expiratory volume % in 1 s
IQR: Standard deviation, interquartile range
IV-fentanyl: Fentanyl-based intravenous analgesia
OR: Odds ratio
PCA: Patient-controlled analgesia
ROC: Receiver operating characteristic
RR: Respiration rate
RRa: Acoustic RR
SpO_2_: Pulse oximetry
